# Amelioration of Chilling Injury by Fucoidan in Cold-Stored Cucumber via Membrane Lipid Metabolism Regulation

**DOI:** 10.3390/foods12020301

**Published:** 2023-01-08

**Authors:** Yupei Zhang, Duo Lin, Ruyu Yan, Yunhe Xu, Mengying Xing, Shuyuan Liao, Chunpeng Wan, Chuying Chen, Liqin Zhu, Wenbin Kai, Jinyin Chen, Zengyu Gan

**Affiliations:** Jiangxi Key Laboratory for Postharvest Technology and Nondestructive Testing of Fruit and Vegetables, Collaborative Innovation Center of Postharvest Key Technology and Quality Safety of Fruit and Vegetables, Jiangxi Agricultural University, Nanchang 330045, China

**Keywords:** cucumber fruit, chilling injury, fucoidan, membrane lipid metabolism

## Abstract

Cucumber fruit is very sensitive to chilling injury, which rapidly depreciates their commodity value. Herein, the effect of fucoidan treatment on cucumber under cold stress were investigated. Fucoidan treatment of cold-stored cucumber alleviated the occurrence of chilling injury, delayed weight loss, lowered electrolyte leakage and respiration rate, and retarded malondialdehyde accumulation. Different from the control fruit, fucoidan treated fruit showed a high level of fatty acid unsaturated content, fatty acid unsaturation, and unsaturation index and increased ω-FDAS activity, along with upregulated expression levels of *CsSAD* and *CsFAD* genes. Fucoidan reduced the phosphatidic acid content and membrane lipid peroxidation, lowered the phospholipase D (PLD) and lipoxygenase (LOX) activity, and downregulated the expression levels of *CsPLD* and *CsLOX* genes. Collectively, fucoidan treatment maintained the integrity of cell membrane in cold-stress cucumbers. The results provide a new prospect for the development of fucoidan as a preservative agent in the low-temperature postharvest storage of cucumbers.

## 1. Introduction

Chilling injury (CI) is one of the most common abiotic stresses in fruit and vegetables encountered during refrigeration. For some fruit and vegetables originating from tropical and subtropical environments with high temperature and high humidity, inappropriate storage temperature or improper operation during cold storage can easily cause chilling damage, resulting in reduced fruit quality [1]. Cucumber is a cold-sensitive vegetable, and low temperatures below 7 °C can cause cell metabolism disorder, resulting in CI [2]. Because cucumber epidermis is thin, cold injury damages the semi-permeability of fruit cell membrane and makes the cytoplasm exude outward. After the fruit is transferred to room temperature, the symptoms of CI develop rapidly, which is manifested as the water-soaked depression of fruit epidermis [3].

CI may cause changes in the cell structure and affect normal physiological and biochemical functions, among which the damage to membrane lipids is the greatest [4,5]. Low temperatures can change the lipid component and fatty acid (FA) unsaturation of membrane lipids, leading to the abnormal functioning of intracellular membranes, which prevents the maintenance of cell homeostasis, and finally causing CI [4,6]. FAs are important components of cell membranes. Different FA contents, components, and their proportions determine the stability of cell membranes. The unsaturated fatty acid (UFA) content decreases during fruit cold storage [7]. FAs commonly found in plant lipids include palmitic acid (16:0), stearic acid (18:0), oleic acid (18:1), linoleic acid (18:2), and linolenic acid (18:3) [6]. Among them, UFAs 18:1, 18:2, and 18:3 play important role in plant stress [8]. The degree of CI in fruit during cold storage is negatively correlated with the content of 18:2 and 18:3 [9]. Saturated fatty acids (SFAs) need to be converted into UFAs through desaturation to perform their functions better. Stearoyl-acyl carrier protein desaturase (SAD) and fatty acid desaturase (FAD) play an important role in FA desaturation. Stearoyl-ACP is one of the main products in the biosynthesis pathway of FAs. SAD can catalyze C16:0 and C18:0 to C16:1 and C18:1, respectively [10], which plays a key role in regulating the unsaturation of FAs in plants and affects the tolerance of plants to low temperatures. After stearic acid is desaturated by SAD to form oleic acid in plants, the subsequent desaturation is completed by FAD. Changes in FAD expression affect plant tolerance to stress by modulating FA unsaturation in cell membrane lipids, altering plant adaptation to extreme temperatures [9].

In cold environment, phospholipids, as one of the important components of plant membrane lipids, can be hydrolyzed or oxidized by phospholipase D (PLD) and lipoxygenase (LOX), resulting in the damage of membrane structure and function [11]. PLD can degrade phospholipid and its activity can directly reflect the degree of hydrolysis of membrane phospholipids. As a key enzyme in FA metabolism, LOX can catalyze the double bond peroxidation of polyunsaturated FAs (such as 18:2 and 18:3), promote the formation of peroxidation products such as malondialdehyde (MDA), and then attack the membrane system and destroy its integrity [12].

There are many methods such as the application of short hot water treatment [13], pre-storage cold acclimation [14], treatment with hydrogen sulfide [15], and dipping in melatonin [16] and chitosan oligosaccharides [17] have been shown to extend the shelf life of refrigerated cucumbers. Fucoidan is a kind of polysaccharide containing fucose and sulfate groups, mainly from brown algae such as seaweed, kelp, wakame, *Sargassum polycystum*, and *Pachyphyllum* algae [18]. Fucoidan is non-toxic and edible, and it has significant antioxidant activity and is a kind of natural antioxidant. At the same time, it also has a variety of immunity properties that can enhance human immunity [19]. Modern studies have shown that fucoidan is also used for preservation of horticultural products. Fucoidan can reduce the loss of mango during postharvest storage and better maintain fruit quality [20]. Fucoidan treatment improves the cold resistance of strawberries by suppressing degradation of total polyphenol and ascorbic acid and suppressing the lowering of antioxidant capacity of strawberries [21,22]. Nevertheless, there is no report on the cold resistance of fucoidan in postharvest cold-stored cucumber. Therefore, in this study, we examined whether fucoidan was related to cold tolerance of cucumber stored under low temperature conditions, specifically focusing on the changes in membrane lipid composition along with the enzymes involved in their metabolism. These findings are expected to provide an in-depth analysis of the mechanism by which fucoidan improves the cold resistance of cucumber fruit.

## 2. Materials and Methods

### 2.1. Plant Materials and Treatments

Cucumber (*Cucumis sativus* cv. Sunstar) fruit were harvested approximately 10 d after flowering at a local orchard in Nanchang, Jiangxi Province, China (28.85° N, 115.55° E, and elevation 35 m). The harvested cucumber fruit were immediately transported to Jiangxi Agricultural University (JXAU) within 2 h, placed at 20 °C to dissipate the field heat, and then washed with distilled water. Fruit of the same size and thickness, no diseases, insect pests, and mechanical damage were divided into two groups for processing. The first group was coated with 15 g L^−1^ fucoidan (Yuanye Bio-Technology, Shanghai, China). The concentration was set by using the previous experimental results (data not shown). The second group was dipped in distilled water and kept as the control. The fruit of two groups were dried naturally at 20 °C, then packed in polyethylene fresh-keeping bags (0.04 mm thick), and stored in a refrigerator at 4 °C for 12 d. Each treatment was repeated three times on 300 fruits. On 0, 3, 6, 9, and 12 d of cold storage, 20 fruit were collected from each replicate, 15 fruit were rewarmed at 20 °C for 2 d to observe the occurrence of CI, and the remaining 5 fruit were taken from mesocarp (pulp tissue) and immediately frozen using liquid nitrogen, and stored at −80 °C.

### 2.2. Measurement of CI, Weight Loss, and Electrolyte Leakage

CI: according to the ratio of CI area (S) to cucumber surface area, CI index of cucumber was divided into 5 levels: Level 0, CI area (S) is 0; Level 1, 0 < S ≤ 25%; Level 2, 25% < S ≤ 50%; Level 3, 50% < S ≤ 75%; Level 4, 75% < S ≤ 100%. CI incidence (%) = (CI fruit/total fruit) × 100%, CI index = Σ(CI level × the number of fruits of this level)/(4 × total number of fruits investigated), with measurements repeated three times.

Weight loss: The weight loss rate was expressed as the percentage of the difference between the initial fruit weight (W_0_) and the fruit weight (W_1_) measured at each time point. Weight loss rate (%) = [(W_0_ − W_1_)/W_0_] × 100%. Four cucumber fruits were weighed, and this process was repeated three times.

Electrolyte leakage: the electrolyte leakage was performed following the procedures reported by Hurr et al. [23], with minor modifications. The tissue discs with uniform thickness and consistent size (diameter: 0.5 cm) were prepared from pulp tissue of cucumber fruit using a hole punch and placed in a triangular cone bottle. The discs were drained of the water with filter paper after washing with double-distilled water (ddH_2_O). Then, the discs were transferred into a 50 mL tube (ten disks per tube), 30 mL of ddH_2_O were added, and electrolyte leakage was measured using a conductivity meter (STARTER 3100C, OHAUS, State of New Jersey, USA) and recorded as C_0_. The discs were shaken for 30 min at 25 °C, and electrolyte leakage was measured again and recorded as C_1_. The sample was heated and boiled for 20 min and cooled to room temperature rapidly; electrolyte leakage was measured and recorded as C_2_. The procedure was repeated three times. Electrolyte leakage was calculated as follows: electrolyte leakage (%) = [(C_1_ − C_0_)/(C_2_ − C_0_)] × 100%.

### 2.3. Measurement of MDA and Respiration Rate

MDA: first, 1.0 g of sample was pulverized with liquid nitrogen, add 5 mL 10% (*w/v*) pre-cooled trichloroacetic acid and fully mix. The mixture was then centrifuged at 10,000× *g* for 20 min at 4 °C. The MDA content was determined using the MDA content detection kit (Solarbio, Beijing, China). The MDA content was expressed as μmol kg^−1^ with three replicates.

The fruit respiration rate was measured using a respiration meter (GHX-3051H, Junfang, Beijing, China). Calibration was performed with 1040 μL·L^−1^ standard CO_2_, the gas flow rate was 0.5 L·min^−1^, and the carrier gas was CO_2_-free air. Three fruits were selected for each measurement, each measurement was repeated three times, and the results were expressed in μg CO_2_·kg^−1^ s^−1^.

### 2.4. Determination of ω-FDAS, PLD, and LOX Activities

ω-FDAS activity was determined using a Plant ω-FADS ELISA kit (Jining, Shanghai, China). The results were shown as U kg^−1^ with three replicates.

PLD activity was determined using a PLD activity detection kit (Jining, Shanghai, China). The results were expressed in U kg^−1^ with three replicates. U represents the amount of enzyme required to hydrolyze PC to produce 1 nmol of choline per gram of sample per minute.

LOX activity was measured using a plant LOX activity detection kit (Solarbio, Beijing, China). The results were expressed in U kg^−1^ with three replicates. U represents the catalytic absorption value varied by 0.001 units per gram of sample per minute.

### 2.5. Determination of Plant Lipids

The sample (20 mg) was homogenized in 1 mL plant lipid extract (methyl tert-butyl ether:methanol = 3:1 [v:v]) and vortexed for 30 min. Then, 300 μL double-distilled water was added, vortexed for 1 min, and then kept at 4 °C for 10 min, centrifuged at 12,000× *g* for 3 min at 4 °C, and then 400 μL supernatant was collected, transferred to 1.5 mL centrifuge tube, and dried completely using a concentrator (Eppendorf, Hamburg, Germany) at 20 °C. Next, 200 μL lipid complex solution (acetonitrile:isopropanol = 1:1 [v:v]) was added to redissolve the sample after being vortexed for 3 min and centrifuged for 3 min at 12,000× *g* at 4 °C. Finally, 120 μL reconstituted solution was collected for liquid chromatography-tandem mass spectrometry (LC-MS/MS) analysis. The detection of plant lipid content was performed at Metware (Metware, Wuhan, China) and the data analysis was completed in the Metware cloud platform (http://cloud.metware.cn/) (accessed on 7 June 2022) [24]. Three biological replicates were measured to verify the accuracy of lipid data. The lipid content was expressed as mmol kg^−1^ of flesh tissue.

The fatty acid unsaturation (UFA/FA) and the unsaturation index (IUFA) are calculated as follows: UFA/FA = Total UFA content/total SFA content, IUFA = Σ(The content of each UFA × the corresponding number of double bonds)/total SFA content.

### 2.6. Gene Expression Assays

RNA extraction and gene expression were measured according to previous study [25]. The quantitative real-time polymerase chain reaction (qRT-PCR) was performed as per the instructions of the 2X M5 HiPer Realtime PCR Supermix (Mei5bio, Beijing, China) on a real-time PCR instrument (CFX96, Bio-Rad, Hercules, CA, USA). The cDNA amplification reaction for each sample was performed in three independent replicates. *CsTUB* was used as the reference gene to quantify the expression of each gene in cucumber fruit via the 2^−ΔΔCt^ method [26]. Gene-expressed primers are listed in Table S1.

### 2.7. Statistical Analysis

SPSS 26.0 software (IBM Inc, Chicago, IL, USA) system was used to analyze the difference significance (Duncan’s multiple range test, *p* < 0.05 or *p* < 0.01). GraphPad Prism 9.0 software (GraphPad Software Inc., San Diego, CA, USA) was used for graphing.

## 3. Results

### 3.1. Fucoidan Treatment Effects on CI of Cucumber Fruit

**Figure 1:**
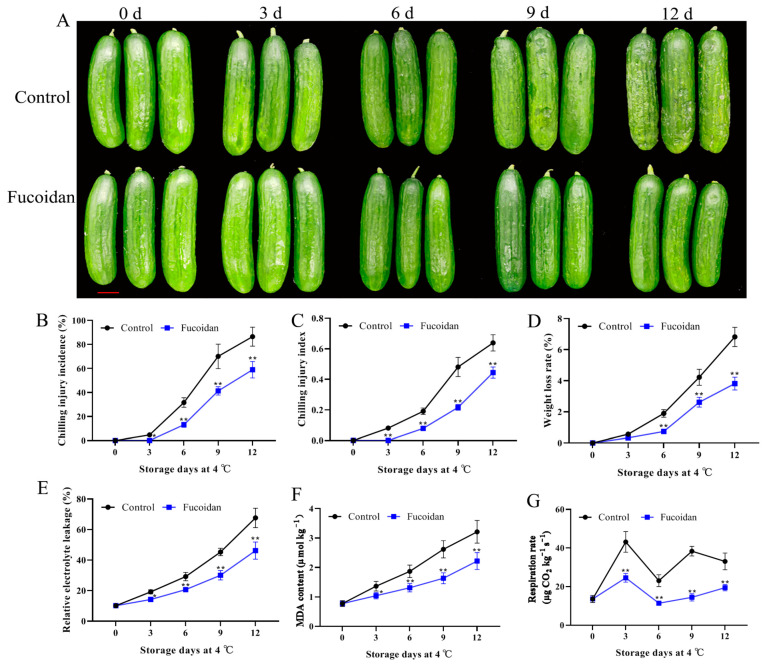
Photograph comparing (**A**), chilling injury incidence (**B**), chilling injury index (**C**), weight loss rate (**D**), relative electrolyte leakage (**E**), MDA content (**F**), and respiration rate (**G**) in cucumber fruit treated with 15 g L^−1^ fucoidan during storage at 4 °C. Cucumber fruit treated with distilled water was kept as the control. Values are marked as mean ± standard deviation (SD) of three replicates. The symbol of * and ** express significant differences at *p* < 0.05 and *p* < 0.01 between fucoidan-treated and control fruit, respectively.

After 3 d of storage at 4 °C, cucumber fruit began to suffer from CI, and the symptoms of CI appeared during the shelf life, which was manifested as the water-soaked depression of fruit epidermis. During the refrigeration period, the degree of CI in control and fucoidan-treated group increased continuously, but the degree of CI of fruit in the fucoidan-treated group was always lower than that of the control group. At the end of the storage period, the CI index of the control group was 0.64, and that of the fucoidan-treated group was 69% of the control group. The results showed that fucoidan treatment delayed the occurrence of CI and reduced the damage caused by low temperatures (Figure 1A–C).

### 3.2. Fucoidan Treatment Effects on Weight Loss, Electrolyte Leakage, MDA Content, and Respiration Rate

The weight loss rate of the cucumber fruit increased continuously with storage time. Fucoidan treatment reduces the weight loss rate of refrigerated cucumber fruit (56% of the control group at the end of storage) (Figure 1D). During the refrigeration period, the cucumber electrolyte leakage increased rapidly, and the electrolyte leakage in the control group was higher than that in the fucoidan-treated group starting from the third day of refrigeration (Figure 1E). The change of MDA content was similar to that of electrolyte leakage. With the increasing degree of CI, the MDA content continued to accumulate; the MDA content in the control group was higher than that in the fucoidan-treated group during the storage period (Figure 1F). The respiration rate of the cucumber fruit in the fucoidan-treated group was lower than that in the control group starting from the third day of refrigeration (Figure 1G).

### 3.3. Fucoidan Treatment Effects on Membrane Lipid Components and Contents

**Figure 2:**
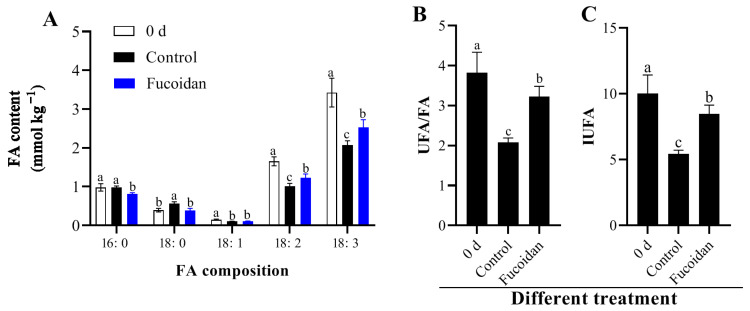
Contents of FA composition (**A**), UFA/FA ratio (**B**), and IUFA (**C**) in cucumber fruit at harvest (0 day) and in fucoidan-treated and control fruit after 12 d of storage at 4 °C. Values are marked as mean ± standard deviation (SD) of three replicates. Bars with same letter mean no significant differences at *p* < 0.05.

Five types of FAs in cucumber fruit at the harvest and 12 d postharvest at 4 °C included two SFAs (palmitic acid [16:0] and stearic acid [18:0]) and three UFAs (oleic acid [18:1], linoleic acid [18:2], and linolenic acid [18:3]). Among them, the palmitic acid content of SFAs was higher, and the UFAs were mainly linolenic acid and linoleic acid. The content of the two UFAs (18:2 and 18:3) decreased after 12 d of refrigeration compared to that at the time of harvest. However, fucoidan treatment can inhibit the decrease in the content of these two UFAs (Figure 2A). Unsaturation reflects the ratio of UFAs to SFAs in cell membrane lipids of cucumber fruit. With the prolongation of refrigeration, the cell membrane lipid unsaturation of cucumber fruit decreased (Figure 2B). On the 12th day of refrigeration, the unsaturation degree of the fucoidan-treated group was higher than that of the control group (Figure 2B,C), indicating that the fucoidan treatment could effectively inhibit the decrease in the unsaturation of cucumber membrane lipids and improve resistance to cold stress.

**Figure 3:**
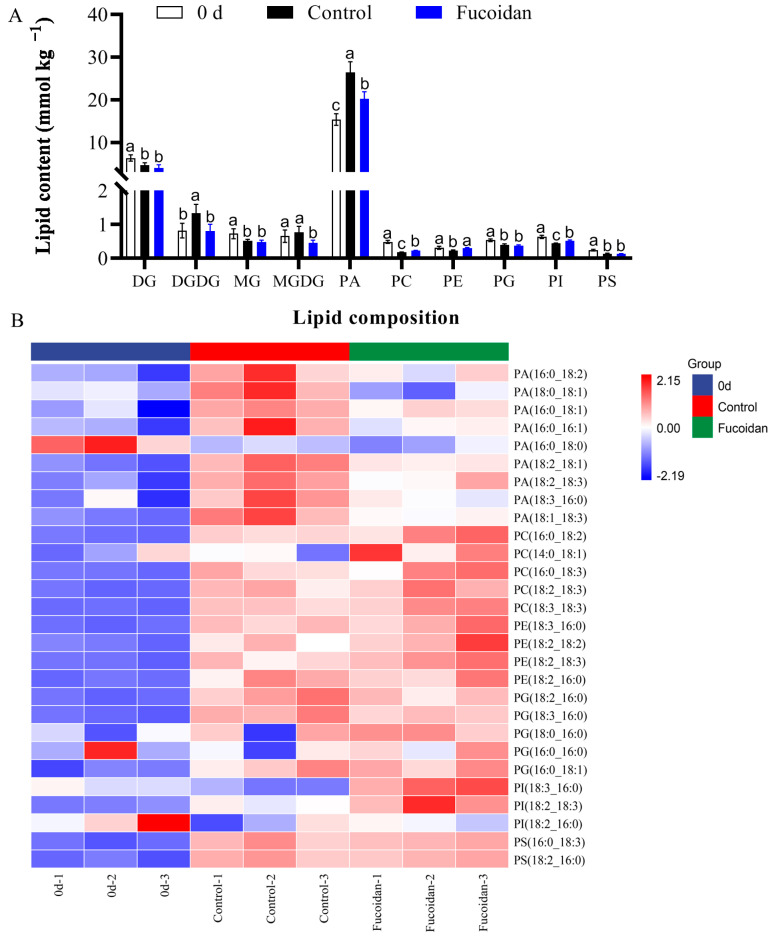
Changes in each phospholipid content (**A**) and each phospholipid species (**B**) in cucumber fruit at harvest (0 day) and in fucoidan-treated and control fruit after 12 d of storage at 4 °C. Values are marked as mean ± standard deviation (SD) of three replicates. Bars with same letter mean no significant differences at *p* < 0.05.

Four types of glycerolipids (diglycerides [DG], monoglycerides [MG], monogalactosyldiacylglycerol [MGDG], and digalactosyldiacylglycerol [DGDG]), and six types of phospholipids (PA, phosphatidylethanolamine [PE], PC, phosphatidylserine [PS], phosphatidylglycerol [PG], and phosphatidylinositol [PI]) were identified in cucumber fruit at harvest and 12 d postharvest at 4 °C (Figure 3A). Among them, the content of DGDG was higher at harvest, but it decreased on the 12th day of refrigeration with no difference between the fucoidan-treated group and the control group. The contents of MGDG and DGDG in the fucoidan-treated group were lower than those of control group, which may be one of the reasons for the difference in the final lipid content. Among the phospholipids, the PA content was much higher than that of the other phospholipid components. The PA content in fucoidan-treated fruit was much lower than those in control fruit on the 12th day of refrigeration. Other identified types of phospholipids except PG and PS increased in fucoidan-treated fruit (Figure 3A).

The molecular species of these six phospholipid components (PA, PC, PE, PG, PI, and PS) were further analyzed. Nine PA molecular species were detected in cucumber fruit. The PA content in fucoidan-treated fruit was lower than that of control fruit, except PA (16:0–18:0), which showed no difference between fucoidan-treated fruit and control fruit on the 12th day of refrigeration. We also identified five PC, four PE, and two PI molecular species, all of which were higher in fucoidan-treated fruit than those of control fruit. Moreover, the contents of five PG and two PS molecular species were not different between the fucoidan-treated and the control fruit (Figure 3B). These results suggest that PA accumulation in cucumber fruit during cold storage may be closely related to the decrease in the PC, PE, and PI contents, and that fucoidan may directly improve the cold tolerance of cucumber fruit by reducing PA accumulation.

### 3.4. Fucoidan Treatment Effects on Enzyme Activities and Related Gene Expression Associated with Membrane Lipids

**Figure 4:**
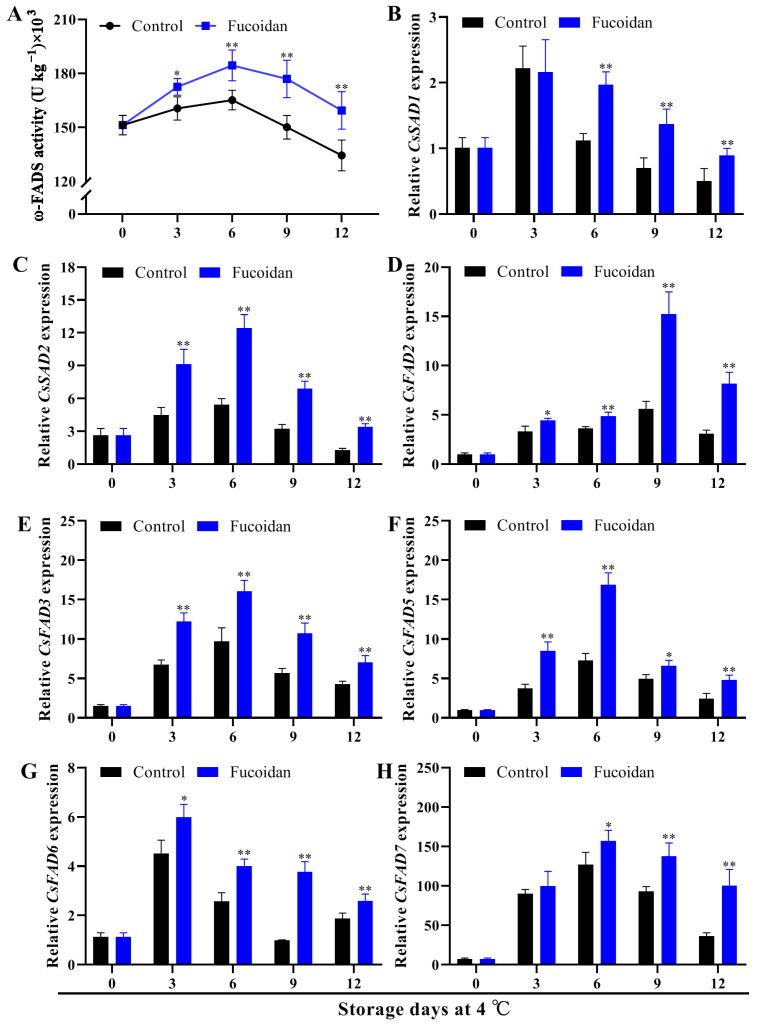
ω-FADS activity (**A**) and gene expression of *CsSAD1* (**B**), *CsSAD2* (**C**), *CsFAD2* (**D**), *CsFAD3* (**E**), *CsFAD5* (**F**), *CsFAD6* (**G**), and *CsFAD7* (**H**) in fucoidan-treated and control cucumber fruit during storage at 4 °C. Values are marked as mean ± standard deviation (SD) of three replicates. Symbols * and ** express significant differences at *p* < 0.05 and *p* < 0.01 between fucoidan-treated and control fruit, respectively.

FAD is a key enzyme in polyunsaturated FA synthesis pathway; thus, we first detected the activity of ω-FADS enzyme. In control cucumber fruit, ω-FADS activity increased during refrigeration and reached the maximum value on day 9. After treatment with fucoidan, the ω-FADS activity showed the same trend as that of the control group, but it was always higher than that of the control group starting from the third day (Figure 4A). To investigate the molecular level of FA metabolism during cold storage of cucumber fruit, we also analyzed the expression of key genes *CsSADs* and *CsFADs* in the UFA synthesis pathway. First, the expressions of *CsSAD1*, *CsSAD2*, *CsFAD2*, *CsFAD3*, *CsFAD5*, *CsFAD6*, and *CsFAD7* increased and then decreased in the control fruit, but fucoidan treatment maintained their expressions at a high level and enhanced this induction, making their transcript levels higher than that in the control group (Figure 4B–H).

**Figure 5:**
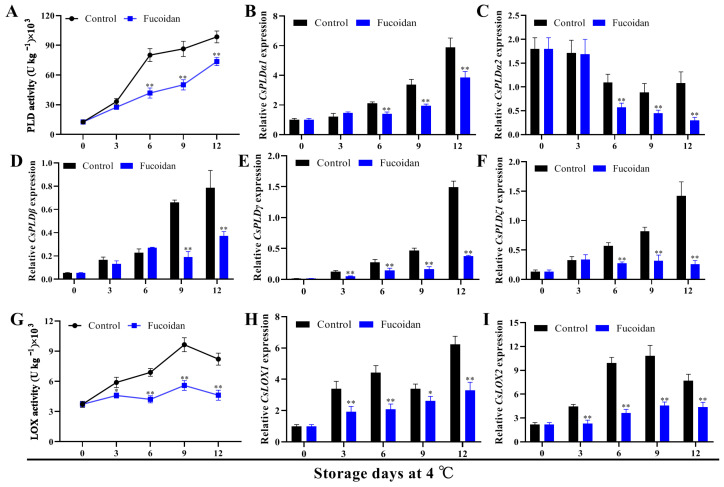
PLD activity (**A**) and gene expression of *CsPLDα1* (**B**), *CsPLDα2* (**C**), *CsPLDβ* (**D**), *CsPLDγ* (**E**), *CsPLDζ1* (**F**), LOX activity (**G**), *CsLOX1* (**H**), and *CsLOX2* (**I**) in fucoidan-treated and control cucumber fruit during storage at 4 °C. Values are marked as mean ± standard deviation (SD) of three replicates. Symbols * and ** express significant differences at *p* < 0.05 and *p* < 0.01 between fucoidan-treated and control fruit, respectively.

PLD and LOX are key enzymes in membrane lipid metabolism, among which PLD can induce phospholipid degradation, whereas LOX can cause membrane lipid peroxidation to damage cytomembrane structure. Both PLD and LOX activities were strongly induced during the refrigeration of cucumber fruit, but the enhancement of their activities was considerably inhibited by fucoidan treatment (Figure 5A,G). We also analyzed the expression of five *CsPLD* and two *CsLOX* genes and observed that the expression of all genes (*CsPLDα1*, *CsPLDβ*, *CsPLDγ*, *CsPLDζ1*, *CsLOX1*, and *CsLOX2*) except *CsPLDα2* increased with refrigeration time, whereas fucoidan treatment inhibited their expression (Figure 5B–F,H,I). Therefore, based on our previous CI occurrence results, we hypothesize that the effect of fucoidan treatment on CI of cucumber fruit may be related to the expression of *CsPLDα1*, *CsPLDβ*, *CsPLDγ*, *CsPLDζ1*, *CsLOX1*, and *CsLOX2*.

## 4. Discussion

Cucumber is sensitive to cold stress, which can easily suffer from cold damage during low-temperature storage and transportation, resulting in water-soaked or sunken spots on the surface of the fruit, rotten and deteriorated pulp, soft tissue, loss of flavor, and severe impact on commodity value [2,3]. In recent years, many exogenous substances and molecules, such as melatonin [16], salicylic acid [27], and glycine betaine [28], have been used as preservatives for cucumbers to prevent the occurrence of CI. Polysaccharide coatings are often used to enhance the antioxidant capacity of horticultural products for preservation. CMC coating maintains the whole quality of pakchoi [29] and delays chilling injury of mandarin [30] and pomegranate fruit [31] by regulating antioxidant capacity. The enhancement of antioxidant enzyme activities was involved in alleviating CI in *Astragalus* polysaccharides treated banana fruit [32]. The stimulation of antioxidant by chitosan contributed to enhanced chilling tolerance in cucumbers [17]. As a nontoxic natural heteropolysaccharide, fucoidan can enhance the body’s immune function by improving the antioxidant defense system in the cardiac, hepatic, and renal tissues [33,34]. As a new preservative, fucoidan has been demonstrated maintain better harvesting quality in some fruit [20,21,22], but whether it can improve the postharvest cold resistance of cucumber fruit remains unknown. The CI index reflects the degree of cucumber CI. Fucoidan reduced the CI index and weight loss rate and showed better appearance and quality of cucumber fruit during refrigeration. Despite the existing hypotheses on the mechanism of CI, the damage of cell membrane structure under cold conditions is the main cause of CI [35]. When cold-sensitive fruits are stored at low temperatures, electrolyte leakage increases considerably and MDA accumulates in large quantities, which promotes the peroxidation of the membrane lipid and leads to the destruction of the membrane structure and increase in membrane permeability, metabolic disorder, and CI [36,37,38]. Fucoidan treatment can inhibit the increase in relative electrolyte leakage and MDA content and alleviate the damage to the cell membrane caused by CI. The respiration rate of fruit increased after being subjected to CI; the higher the respiration rate, the more severe the CI, which may be a response of fruit self-protection [39]. In this study, exogenous fucoidan treatment inhibited the respiration rate of cucumber and thus the consumption of organic matter in fruit caused by respiration.

The unsaturation of FAs affects the fluidity of membrane lipids and thus the cold tolerance of plants [40]. Both IUFA and UFA/FA decreased when the fruit were subjected to CI. In this study, the UFA content (18:2 and 18:3), IUFA, and UFA/FA of cucumber fruit decreased considerably after 12 d of cold storage, which was similar to the results of pepper fruit [9], peach fruit [41], and zucchini fruit [42]. However, exogenous fucoidan treatment ameliorated this reduction in cucumber fruit. FA desaturase is a key enzyme for polyunsaturated FA synthesis pathways, which catalyzes the formation of double bonds at specific positions on the FA chain. In pepper fruit, cold reduced total FAD activity and damage the desaturation of FA [37]. We showed that ω-FADS activity increased first and decreased at the end during the cold storage of cucumber, which may be related to the expression of related genes. *SAD* and *FAD* are key genes for the synthesis of UFAs, and their relationship with cold tolerance in plants has been extensively reported [9,36,37]. In many horticultural plants, gene expression of *FAD* was positively correlated with chilling resistance [4,37]. Under cold stress, the expression of most cucumber *FAD* genes was up-regulated [43]. In this study, the expression of *SADs* (*CsSAD1/2*), ω3 subfamily *FADs* (*CsFAD3/7*), ω6 subfamily *FADs* (*CsFAD2/6*), or other *FADs* (*CsFAD5*) was induced by low temperatures in the early stage of storage, and the expression decreased in the later stage, which is related to the activity of ω-FADS. These results indicate that under low temperature stress in cucumber fruit, the expression of *SAD* and *FAD* genes was induced, and UFAs were increased to alleviate the symptoms of CI. However, since the upregulation of *SAD* and *FAD* in the later stage is far less than that of key genes that promote membrane lipid degradation, such as *PLD* and *LOX*, the final failure to maintain the balance of membrane lipid components leads to the occurrence of CI. After fucoidan treatment, the symptoms of CI were alleviated in the late storage period, the expression of *SADs* and *FADs* increased, and the UFA 18:2 and 18:3 contents increased. These results may help enhance the cold resistance of cucumber.

Excessive accumulation of PA by cold stress can destroy the integrity and stability of cell membrane. In peppers [38,44], peaches [4,45], pears [46], and bananas [47], cold stress induced PA accumulation results in CI, which corresponds to our findings for cucumber fruit. However, fucoidan treatment inhibited the PA accumulation induced by cold stress, retaining the stability of cell membrane and enhancing the cold resistance of cucumber. PLD is a key enzyme for PA synthesis from phospholipid metabolism. The activity and expression of PLD are induced by CI [38,44]. In the *AtPLDα* mutant, the PC content drastically decreased after cold treatment, and the cold tolerance was stronger, indicating that inhibiting the expression of *PLD* may alleviate CI [48]. In this study, we measured PLD activity and the expression of five *PLD* genes and showed that the PLD activity and all *PLD* genes except *CsPLDα2* increased with refrigeration time, which were consistent with the cold-induced changes in PA content. Moreover, fucoidan inhibits the expression of PLD and maintains the stability of cell membrane, reducing the accumulation of PA content, thereby improving the cold tolerance of cucumber fruit.

LOX catalyzes the peroxidation of UFAs, reduces the UFAs of membrane lipids, and intensifies the damage to plant cell membrane structure and function. LOX is considered a key factor in the metabolism of UFAs and membrane lipid peroxidation [49]. Under cold stress, enhanced LOX activity is accompanied by an increase in cell membrane permeability, resulting in CI [11,37]. Therefore, inhibiting LOX enzyme activity has a strong effect on maintaining the cell membrane integrity and improving the fruit cold tolerance. Many exogenous substances, such as melatonin [11,41] and salicylic acid [37], can inhibit LOX activity and related gene expression in fruit, thereby improving cold resistance. In our study, fucoidan treatment suppressed the LOX activity and the expression of *CsLOX1/2* during cold storage and alleviated the CI of cucumber fruit.

## 5. Conclusions

**Figure 6:**
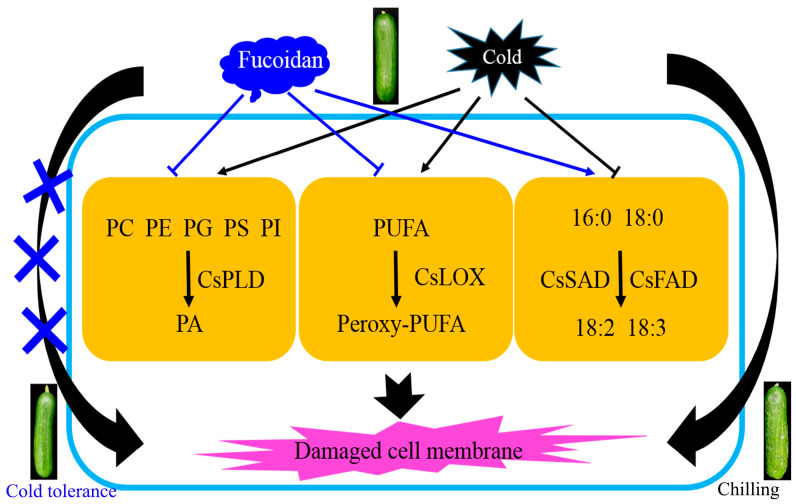
Amelioration of chilling injury in cold-stored cucumber fruit by potential mechanism of fucoidan acting on membrane lipid metabolism. PC, phosphatidylcholine; PE, phosphatidylethanolamine; PG, phosphatidylglycerol; PS, phosphatidylserine; PI, phosphatidylinositol; PA, phosphatidic acid; PUFA, polyunsaturated fatty acid; 16:0, palmitic acid; 18:0, stearic acid; 18:2, linoleic acid; 18:3, linolenic acid.

The occurrence of CI in cucumber fruit during storage at 4 °C was effectively alleviated via fucoidan treatment. The alleviation of CI by fucoidan was involved in the regulation of membrane lipid metabolism. Fucoidan treatment enhanced the activity of ω-FADS, increased the index of UFAs, inhibited the activities of PLD and LOX, reduced the accumulation of PA and the degree of membrane lipid peroxidation, alleviated the damage to cell membrane caused by CI, maintained the cell membrane integrity, and improved the cold resistance of cucumber fruit (Figure 6). Therefore, we speculated that fucoidan treatment can be considered a new reference to optimize the low-temperature storage condition and minimize the detrimental effects induced by CI in cucumber fruits.

## Data Availability

The data generated from the study are clearly presented and discussed in the manuscript.

## Supplementary Materials

The following supporting information can be downloaded at: https://www.mdpi.com/article/10.3390/foods12020301/s1, Table S1: Primers for gene expression.
